# Inactivation of ID4 promotes a CRPC phenotype with constitutive AR activation through FKBP52

**DOI:** 10.1002/1878-0261.12028

**Published:** 2017-03-02

**Authors:** Jugal Bharat Joshi, Divya Patel, Derrick J. Morton, Pankaj Sharma, Jin Zou, Dhanushka Hewa Bostanthirige, Yamini Gorantla, Peri Nagappan, Shravan Kumar Komaragiri, Jeffrey C. Sivils, Huan Xie, Ravi Palaniappan, Guangdi Wang, Marc B. Cox, Jaideep Chaudhary

**Affiliations:** ^1^ Center for Cancer Research and Therapeutic Development Clark Atlanta University GA USA; ^2^ College of Pharmacy Mercer University Atlanta GA USA; ^3^ Department of Biological Sciences Border Biomedical Research Center University of Texas at El Paso TX USA; ^4^ College of Pharmacy and Health Sciences Texas Southern University Houston TX USA; ^5^ Department of Chemistry RCMI Cancer Research Center Xavier University of Louisiana New Orleans LA USA

**Keywords:** androgen receptor, castration‐resistant, FKBP51, FKBP52, HLH, Hsp27, Hsp90, ID4, MJC13, prostate cancer, PSA

## Abstract

Castration‐resistant prostate cancer (CRPC) is the emergence of prostate cancer cells that have adapted to the androgen‐depleted environment of the prostate. In recent years, targeting multiple chaperones and co‐chaperones (e.g., Hsp27, FKBP52) that promote androgen receptor (AR) signaling and/or novel AR regulatory mechanisms have emerged as promising alternative treatments for CRPC. We have shown that inactivation of inhibitor of differentiation 4 (ID4), a dominant‐negative helix loop helix protein, promotes *de novo* steroidogenesis and CRPC with a gene expression signature that resembles constitutive AR activity in castrated mice. In this study, we investigated the underlying mechanism through which loss of ID4 potentiates AR signaling. Proteomic analysis between prostate cancer cell line LNCaP (L+ns) and LNCaP lacking ID4 (L(−)ID4) revealed elevated levels of Hsp27 and FKBP52, suggesting a role for these AR‐associated co‐chaperones in promoting constitutively active AR signaling in L(−)ID4 cells. Interestingly, protein interaction studies demonstrated a direct interaction between ID4 and the 52‐kDa FK506‐binding protein (FKBP52) *in vitro*, but not with AR. An increase in FKBP52‐dependent AR transcriptional activity was observed in L(−)ID4 cells. Moreover, pharmacological inhibition of FKBP52‐AR signaling, by treatment with MJC13, attenuated the tumor growth, weight, and volume in L(−)ID4 xenografts. Together, our results demonstrate that ID4 selectively regulates AR activity through direct interaction with FKBP52, and its loss, promotes CRPC through FKBP52‐mediated AR signaling.

**Abbreviations**
ADT: androgen deprivation therapy
AR: androgen receptor
BF3: binding function 3
bHLH: basic helix loop helix
ChIP: chromatin immunoprecipitation
Co‐IP: co‐immunoprecipitation
CRPC: castration‐resistant prostate cancer
csFBS: charcoal‐stripped FBS
ELISA: enzyme‐linked immunosorbent assay
FKBP52: FK506‐binding protein 4
HLH: helix loop helix
Hsp90: heat shock protein 90
ICC: immunocytochemistry
ID4: inhibitor of differentiation 4
IHC: immunohistochemistry
LBD: ligand binding domain
PCa: prostate cancer
PPIase: peptidyl‐prolyl cis‐trans isomerase
PRAD: prostate cancer adenocarcinoma
qRT‐PCR: quantitative RT‐PCR
TCGA: The Cancer Genome Atlas
TPR: tetratricopeptide repeat

## 1 Introduction

The androgen receptor (AR) is a nuclear receptor transcription factor required for normal prostate development and prostate cancer pathogenesis (Zhang *et al*., 2016). Until now, androgen deprivation therapy (ADT) that inhibits AR signaling has been the frontline therapy for the treatment of patients with hormone‐sensitive metastatic prostate cancer (Shi *et al*., 2015). Although ADT is initially effective, patients invariably relapse and their tumors progress to castration‐resistant prostate cancer (CRPC) (Egan *et al*., 2014). As a result, the clinically available therapeutic options including androgen deprivation, classic AR antagonists, and inhibitors of *de novo* steroidogenesis ultimately fail (Yamaoka *et al*., 2010). Because CRPC is commonly associated with aberrant AR signaling that is sufficient to overcome ADT (Polkinghorn *et al*., 2013), blocking AR signaling through multiple mechanisms remains a valid therapeutic strategy (Heinlein and Chang, 2004; Sung and Cheung, 2014). Thus, there is a need for the identification, characterization, and therapeutic targeting of novel molecular mechanisms and distinct regulatory proteins that promote prolonged AR activation in the advanced stages of prostate cancer.

In general, folding of AR to a mature hormone binding conformation is a highly ordered, dynamic assembly of heteromeric complexes that involves multiple chaperone and co‐chaperone components (Smith and Toft, 2008), most of which represent potential targets for the treatment of prostate cancer (Cano *et al*., 2013). The final mature complex in which the receptor is capable of high‐affinity hormone binding includes heat shock protein 90 (Hsp90), the p23 co‐chaperone, and the 52‐kDa FK506‐binding protein (FKBP52, also termed FKBP4) (Cheung‐Flynn *et al*., 2005; Riggs *et al*., 2003; Tranguch *et al*., 2005). FKBP52 belongs to a subclass of tetratricopeptide repeat (TPR)‐containing co‐chaperone proteins that are diverse regulators of steroid hormone receptor signaling, including the regulation of receptor maturation, hormone binding, and nuclear translocation (Davies and Sanchez, 2005; Storer *et al*., 2011). Given the critical role of FKBP52 in AR signaling in cellular and whole animal models, both *in vitro* and *in vivo*, FKBP52 has emerged as a potential target for the treatment of prostate cancer (De Leon *et al*., 2011; Guy *et al*., 2015; Storer Samaniego *et al*., 2015; Yong *et al*., 2007). FKBP52 association with receptor–Hsp90 complexes results in the enhancement of hormone binding (Davies *et al*., 2005; Riggs *et al*., 2003, 2007), yet the mechanism by which this occurs is still unknown. Although peptidyl‐prolyl cis‐trans isomerase (PPIase) enzymatic activity is not required for FKBP52 potentiation of AR activity, the PPIase domain (FK1) is essential. Moreover, the FKBP52 proline‐rich loop that overhangs the PPIase catalytic pocket within the FK1 domain represents an interaction surface that transiently contacts the receptor hormone binding domain within the AR–chaperone complex (De Leon *et al*., 2011; Storer Samaniego *et al*., 2015). In the recent years, the AR binding function 3 (BF3) surface has been identified as the likely site of FKBP52 regulation. MJC13 is thought to specifically inhibit FKBP52‐regulated AR activity by binding to the AR‐BF3 surface located within the receptor ligand binding domain (LBD) (De Leon *et al*., 2011; Storer Samaniego *et al*., 2015).

Inhibitor of differentiation 4 (ID4), a helix loop helix (HLH) protein, is a dominant‐negative transcriptional regulator of basic HLH (bHLH) family of transcription factors (Benezra *et al*., 1990). Previous studies demonstrated that ID4 is highly expressed in normal ductal epithelial cells of the prostate, whereas ID4 expression is progressively lost with increasing stage of the prostate cancer due to promoter hypermethylation (Carey *et al*., 2009; Sharma *et al*., 2012). Our previous developmental studies on prostate glands from ID4^−/−^ mice demonstrated a significant attenuation of AR transcriptional activity, further resulting in decreased expression of NKX3.1, an important androgen‐regulated gene required for normal prostate development (Sharma *et al*., 2013). Furthermore, our recent studies also suggested that loss of ID4, frequently observed in PCa, promotes CRPC through constitutive AR activation (Patel *et al*., 2014). Collective observations based on our studies (Asirvatham *et al*., 2006; Carey *et al*., 2009; Chaudhary *et al*., 2005; Patel *et al*., 2014, 2015; Sharma *et al*., 2012, 2013) led us to hypothesize that ID4 could be involved in selectively regulating AR activity through FKBP52 in prostate cancer. The present study was aimed to determine the underlying molecular mechanism through which loss of ID4 promotes a CRPC phenotype with increased AR transcriptional activity. Here, we report that cross‐talk between ID4, FKBP52, and AR regulates AR transcriptional activity, whereas loss of ID4 potentiates FKBP52–AR interaction. In summary, we propose that inactivation of ID4 promotes transcriptional activation of androgen receptor in the castration‐resistant environment, a condition of significant clinical interest.

## 2 Materials and methods

### 2.1 Cell lines and ID4 silencing

The PCa cell line LNCaP was used to stably silence ID4 using gene‐specific short hairpin RNA retroviral vectors (L(−)ID4 cells) as described in our previous study (Knowell *et al*., 2013). The cells transfected with nonsilencing short hairpin RNA (L+ns) were used as controls. For measuring androgen responses, the cells were cultured in charcoal‐stripped FBS (csFBS) for 24 h and subsequently treated with vehicle or 10 nm synthetic androgen R1881 and/or 30 μm bicalutamide (Casodex; a gift from AstraZeneca, Wilmington, DE, USA) for 24 h. Cells were cultured at 37 °C in a fully humidified atmosphere containing 5% CO_2_.

### 2.2 Co‐immunoprecipitation and quantitative mass spectrometry analysis

Co‐immunoprecipitation analysis using ID4‐specific antibody on L+ns cells was performed with the help of protein A coupled to magnetic beads (Protein A Mag beads; GenScript, Piscataway, NJ, USA) as per the manufacturer's instructions. Next, ID4 interacting partner's co‐immunoprecipitated with ID4 was identified via LC‐MS/MS analysis on LTQ‐Orbitrap.

#### 2.2.1 LC‐MS/MS analysis on LTQ‐Orbitrap

Peptides were analyzed on an LTQ‐Orbitrap XL (Thermo Scientific, Waltham, MA, USA) instrument interfaced with an Ultimate 3000 Dionex LC system (Dionex, Sunnyvale, CA, USA) by using high mass resolution to identify peptides and high‐energy collision dissociation (HCD) to quantify reporter ions. The reverse‐phase HPLC system consisted of a peptide Cap‐Trap cartridge (0.5 by 2 mm) (Michrom BioResources, Auburn, CA, USA) and a prepacked Bio Basic C18 PicoFrit analytical column (75 μm inner diameter × 15 cm length; New Objective, Woburn, MA, USA) fitted with a FortisTip emitter tip. Samples were loaded onto the trap cartridge and washed with mobile phase A (98% H_2_O, 2% acetonitrile, and 0.1% formic acid) for concentration and desalting. Subsequently, peptides were eluted for more than 3 h from the analytical column via the trap cartridge by using a linear gradient of 6–100% mobile phase B (20% H_2_O, 80% acetonitrile, and 0.1% formic acid) at a flow rate of 0.3 μL·min^−1^ by using the following gradients: 6% B for 5 min, 6–60% B for 125 min, 60–100% B for 5 min, 100% B for 5 min, 100–6% B for 2 min, and 6% B for 38 min.

The Orbitrap mass spectrometer was operated in a data‐dependent mode in which each full MS scan (60 000 resolving power) was followed by six MS/MS scans where the three most abundant molecular ions were dynamically selected and fragmented via collision‐induced dissociation by using a normalized collision energy of 35%, and the same three molecular ions were also scanned by HCD‐MS^2^ with collision energy of 45%. MS scans were acquired in profile mode and MS/MS scans in centroid mode. LTQ‐Orbitrap settings were as follows: spray voltage 2.0 kV, one microscan for MS^1^ scans at 60 000 resolution (fwhm at *m*/*z* 400), microscans for MS^2^ scans at 7500 resolution (fwhm at *m*/*z* 400); full MS mass range, *m*/*z* 400–1400; MS/MS mass range, *m*/*z* 100–2000. The ‘FT master scan preview mode’, ‘charge state screening’, ‘monoisotopic precursor selection’, and ‘charge state rejection’ were enabled so that only the 2+, 3+, and 4+ ions were selected and fragmented by collision‐induced dissociation and HCD.

### 2.3 Poly‐histidine pull‐down assay

Poly‐histidine pull‐down assays using wild‐type His6‐FKBP52 (Storer Samaniego *et al*., 2015), full‐length ID4, and truncated ID4 constructs including ID4S73A (ID4 HLH mutant) and/or ID4∆A mutant (ID4 in which the alanine tract was deleted) (Sharma *et al*., 2015) were performed as per the manufacturer's instructions (Pierce™ Pull‐Down Poly Histidine Protein‐Protein Interaction kit; Thermo Scientific, IL, USA).

### 2.4 GST‐AR pull‐down assay

GST pull‐down assays using GST‐tagged AR, His6‐FKBP52, and recombinant ID4 proteins were performed as per the manufacturer's instructions (Pierce™ Pull‐Down GST Protein‐Protein Interaction kit; Thermo Scientific). GST‐tagged AR plasmids were provided as a generous gift from Dr Amina Zoubeidi (University of British Columbia, Vancouver, Canada).

### 2.5 GST‐ID4 pull‐down assay

Glutathione S‐transferase (pReceiver‐BO4) fused in frame to protein‐coding region of human ID4 (GST‐ID4) plasmid was custom‐synthesized by Genecopoeia. Full‐length GST‐ID4 and truncated GST‐ID4 fusion proteins including ID4S73A (ID4 HLH mutant) and/or ID4∆A mutant (ID4 in which the alanine tract was deleted) were expressed and purified as per the manufacturer's instructions. Plasmids were transformed into BL21 (DE3)‐competent cells (Novagen, Darmstadt, Germany). Protein expression in the freshly grown bacterial cultures at 37 °C was then induced by the addition of IPTG (1 mm) at 30 °C. Four hours after induction, the BL21 (DE3) cells were centrifuged. The respective pellets were lysed at room temperature for 15 min in B‐PER (Thermo Scientific, Inc.) with DNase (three units) and lysozyme (100 μg). The lysates were then centrifuged at 12 857 ***g*** for 10–15 min at 4 °C. Next, the pellets were washed extensively with 1× PBS and boiled in SDS sample buffer. The GST‐ID4 column‐bound proteins using LNCaP and DU145 whole‐cell lysates were size‐fractionated on 4–20% SDS/polyacrylamide gel and then subjected to immunoblotting analysis using protein‐specific antibodies (Supporting information). The LAS 3000 imager (Fuji, FujiFilm LAS‐3000, Stamford, CT, USA) was used to capture the images.

### 2.6 Immunoblot and co‐immunoprecipitation analysis

Cellular, nuclear, and cytoplasmic proteins were prepared from cultured prostate cancer cell lines using M‐PER and N‐PER kits (Thermo Scientific). Twenty microgram of total protein was size‐fractionated on 4–20% SDS/polyacrylamide gel. The SDS/PAGE was subsequently blotted onto a nitrocellulose membrane (Whatman, St. Louis, MO, USA) and subjected to western blot analysis using protein‐specific antibodies (Supporting information). After washing with 1× PBS, 0.5% Tween 20, the membranes were incubated with horseradish peroxidase (HRP)‐coupled secondary antibody against rabbit IgG and visualized using the Super Signal West Dura Extended Duration Substrate (Thermo Scientific). The LAS 3000 imager (Fuji) and image quant software were used to capture and quantify the images.

To detect the protein–protein interactions, co‐immunoprecipitation was performed using protein A coupled to magnetic beads (Protein A Mag beads; GenScript) as per the manufacturer's instructions. Briefly, protein‐specific IgG (anti‐FKBP52, or anti‐AR, Supporting information) was first immobilized to Protein A Mag Beads by incubating overnight at 4 °C. To minimize the co‐elution of IgG following immunoprecipitation, the immobilized IgG on Protein A Mag beads was cross‐linked in the presence of 20 mm dimethyl pimelimidate dihydrochloride (DMP) in 0.2 m triethanolamine, pH 8.2, washed twice in Tris (50 mm Tris, pH 7.5) and PBS followed by final resuspension and storage in PBS. The cross‐linked protein‐specific IgG/Protein A Mag beads were incubated overnight (4 °C) with freshly extracted total cellular proteins (500 μg·mL^−1^). The complex was then eluted with 0.1 m glycine (pH 2–3) after appropriate washing with PBS and neutralized by adding 10 μL of neutralization buffer (1 m Tris, pH 8.5) per 100 μL of elution buffer.

### 2.7 Immunocytochemistry

Cells were grown on glass chamber slides up to 75% confluency in a six‐well plate. Twenty‐four hours after plating, the complete medium with 10% FBS was replaced with 10% charcoal‐stripped fetal bovine serum (csFBS) media. The slides were then washed with PBS (3×) and fixed in ice‐cold methanol for 10 min at room temperature and stored at 4 °C until further use. Before use, the slides were equilibrated at room temperature, washed with PBS (5 min × 3), blocked with 1% BSA in PBST for 30 min at room temperature, and incubated overnight (4 °C) with protein‐specific antibodies (1% BSA in PBST, Supporting information). The slides were then washed in PBS and incubated with secondary antibody with fluorochrome conjugated to DyLight (Supporting information) in 1% BSA for 1 h at room temperature in dark. The slides were subsequently washed again and stained in DAPI (1 μg·mL^−1^) for 1 min and mounted with glycerol. Images were acquired by Zeiss fluorescence microscope through axiovision software.

### 2.8 Immunohistochemistry

Slides were processed through standard protocols. Following antigen retrieval (autoclave in 0.01 m sodium citrate buffer, pH 6.0, at 121 °C/20 psi for 30 min), the peroxidase activity was blocked in 3% H_2_O_2_ and nonspecific binding sites blocked in 10% goat serum. The blocked sections were incubated overnight at 4 °C with protein‐specific primary antibodies followed by incubation with secondary antibody (Supporting information) for 1 h. The slides were stained with DAB for 2 min, counterstained with hematoxylin, mounted with immunomount (Thermo Scientific), and examined, and photomicrographs were taken using the Zeiss microscope with an axiovision version 4.8 imaging system. All the antibodies were monoreactive; that is, a single reactive band was observed in western blot using whole‐cell lysates from prostate cancer cell lines LNCaP, DU1545, and PC3. Nonspecific binding of the secondary antibodies was evaluated using respective normal IgGs (data not shown).

### 2.9 AR protein stability assay

L+ns and L(−)ID4 cells were grown in RPMI 1640 medium containing 10% fetal bovine serum up to 75% confluency. Cells were treated with cycloheximide (100 μg·mL^−1^) for 0, 6, 12, 24, 30, and 36 h, followed by the preparation of whole‐cell lysates. AR protein levels were determined by western blot analysis with specific antibody directed against AR and normalized to GAPDH (loading control).

### 2.10 RNA preparation and quantitative RT‐PCR analysis

Total RNA from L+ns and L(−)ID4 cells was isolated by an E.Z.N.A. DNA/RNA kit (Omega Bio‐Tek, Norcross, GA, USA). RNA (2 μg) isolated from cells was then reverse‐transcribed in a final volume of 25 μL as per standard protocols (Asirvatham *et al*., 2006; Sharma *et al*., 2013). Reverse‐transcribed RNA was used to performreal‐time quantitative RT‐PCR analysis (qRT‐PCR) analysis using gene‐specific primers (Supporting information).

### 2.11 Chromatin immunoprecipitation assay

Chromatin immunoprecipitation was performed using the chromatin immunoprecipitation (ChIP) assay kit (Millipore, Billerica, MD, USA) as per the manufacturer's instructions. Briefly, L+ns and L‐ID4 cells were grown in RPMI‐1640 medium with 10% FBS (charcoal‐stripped) for 3 days and treated with R1881 (10 nm) or vehicle for 24 h. The chromatin (total DNA) extracted from cells was sheared (Covaris S220; Covaris Inc., Woburn, MA, USA), subjected to immunoprecipitation with AR, normal IgG or RNA Pol II antibodies at 4 °C overnight, reverse‐cross‐linked, and then subjected to qRT‐PCR analysis in the Eco Real‐Time PCR system (Illumina, San Diego, CA, USA). The previously published ChiP primer sets spanning the consensus androgen response element sites in the promoters of PSA (Louie *et al*., 2003), FKBP51 (Makkonen *et al*., 2009), TMPRSS2 (Menon *et al*., 2010), and ETV1 (Cai *et al*., 2007) were used (Supporting information).

### 2.12 Luciferase reporter assays

Cells were plated in a 96‐well plate at a density of 2.5 × 10^4^ cells/well. After the cells attached, they were transiently transfected by mixing either ARR3‐luciferase or PSA luciferase with pGL4.74 plasmid (*hRluc/TK*: Renilla luciferase; Promega, Madison, WI, USA) DNA in a 9 : 1 ratio with FuGENE HD transfection reagent (Promega) in a final volume of 100 μL of RPMI 1640 medium and incubated for 15 min at room temperature. The transfection mix was then added to the cells followed by the addition of R1881 (10 nm) or vehicle after 4 h. After a total of 24 h, the cells were assayed for firefly and Renilla luciferase activities using the Dual‐Glo Luciferase reporter assay system (Promega) in LUMIstar OPTIMA (MHG Labtech, BMG LABTECH, Cary, NC, USA). The results were normalized for the internal Renilla luciferase control.

### 2.13 Proteomic analysis

Quantitative proteomic analysis of L+ns and L(−)ID4 cells was performed according to the standard procedure.

#### 2.13.1 Cell analysis

L+ns and L(−)ID4 cell pellets obtained from one T‐75 flask each were lysed with 1 mL of M‐PER (Thermo Scientific) plus 10 μL of phosphatase inhibitor (Thermo Scientific) and 10 μL of protease inhibitor (Thermo Scientific), followed by sonication and centrifugation at 16 000 ***g*** for 5 min to remove insoluble material from the crude lysate. The supernatant was collected and the concentration of total protein was determined with the BCA protein assay kit (Pierce Biotechnology, Rockford, IL, USA). The protein concentration was measured to be in the range of 1–2 mg·mL^−1^.

#### 2.13.2 Reduction, alkylation, and trypsin digestion

L+ns and L(−)ID4 cell lyses were triplicated with 100 μg of total proteins. Aliquots of 100 μg of each protein sample were added into 100 μL of 200 mm triethyl ammonium bicarbonate (TEAB) (Sigma, St. Louis, MO, USA). Reduction was performed by adding 5 μL of 200 mm Tris (2‐carboxyethyl) phosphine (TCEP) (Sigma) to each replicate followed by 1‐h incubation at 55 °C. Next, alkylation was carried out by adding 5 μL of 375 mm iodoacetamide (Bio‐Rad Laboratories, Hercules, CA, USA) to each replicate followed by 30‐min incubation in dark at room temperature. After alkylation, 1 mL of prechilled acetone was added and precipitation was allowed to proceed overnight at −20 °C. The acetone‐precipitated protein pellets were suspended into a 100 μL solution of 200 mm TEAB, and then, 2.5 μg of sequencing‐grade modified trypsin (Promega Corp.) was added to digest each replicate overnight at 37 °C.

#### 2.13.3 Isobaric labeling with TMT

Next, tandem mass tags TMT6 (Thermo Scientific) with different molecular weights (126–131 Da) were applied as isobaric tags for quantification analysis. As per the manufacturer's instructions, each digested sample was individually labeled with a different isobaric tag. Three 100‐μg aliquots of digested peptides of L+ns were labeled with TMT126, 127, and 128, whereas three 100‐μg aliquots of digested peptides of L(−)ID4 were labeled with TMT‐129, 130, and 131, respectively. The labeling reaction was quenched with 5% hydroxylamine. Finally, the labeled peptide mixtures were combined at equal ratios.

#### 2.13.4 Fractionation of labeled peptide mixture using a strong cation‐exchange column

The combined TMT‐labeled peptide mixture was fractionated with a strong cation‐exchange SCX column (Thermo Scientific) on a Shimadzu Ultra‐Fast Liquid Chromatography (UFLC) equipped with an ultraviolet detector (Shimadzu, Columbia, MD, USA). Mobile phase consisted of buffer A (5 mm KH_2_PO_4_, 25% acetonitrile, and pH 2.8) and buffer B (buffer A plus 350 mm KCl). The column was equilibrated with buffer A for 30 min before sample injection. The mobile‐phase gradient was set as follows, at a flow rate of 1.0 mL·min^−1^: (a) 0–10 min: 0% buffer B; (b) 10–40 min: 0–25% buffer B; (c) 40–45 min: 25–100% buffer B; (d) 45–50 min: 100% buffer B; (e) 50–60 min: 100–0% buffer B; and (f) 60–90 min: 0% buffer B. In all, 60 fractions were initially collected, lyophilized, and combined into 14 final fractions based on SCX chromatogram peaks.

#### 2.13.5 Desalination of fractionated samples

A C18 solid‐phase extraction (SPE) column (Hyper‐Sep SPE Columns, Thermo Scientific) was used to desalt all collected fractions. The combined 14 fractions were each adjusted to a final volume of 1 mL containing 0.25% (v/v in water) trifluoroacetic acid (TFA). The C18 SPE column was conditioned before use by filling with 1 mL acetonitrile and allowing the solvent to pass through the media slowly (3 min). The column was then rinsed three times with 1 mL 0.25% TFA solution. The fractions were loaded on to the top of the SPE cartridge column slowly and reloaded once again to the column to decrease lost peptide during the column binding. Columns were washed four times with 1 mL 0.25% TFA aliquots before the peptides were eluted three times, each with 400 μL of 80% acetonitrile/0.1% formic acid (aqueous). All of the eluted samples were then lyophilized for the LC‐MS/MS.

#### 2.13.6 LC‐MS/MS analysis on LTQ‐Orbitrap

Peptides were analyzed on an LTQ‐Orbitrap XL (Thermo Scientific, Waltham, MA, USA) instrument interfaced with an Ultimate 3000 Dionex LC system (Dionex) by using high mass resolution to identify peptides and high‐energy collision dissociation (HCD) to quantify reporter ions. The reverse‐phase HPLC system consisted of a peptide Cap‐Trap cartridge (0.5 by 2 mm) (Michrom BioResources) and a prepacked Bio Basic C18 PicoFrit analytical column (75 μm inner diameter × 15 cm length; New Objective) fitted with a FortisTip emitter tip. Samples were loaded onto the trap cartridge and washed with mobile phase A (98% H_2_O, 2% acetonitrile, and 0.1% formic acid) for concentration and desalting. Subsequently, peptides were eluted for more than 3 h from the analytical column via the trap cartridge by using a linear gradient of 6–100% mobile phase B (20% H_2_O, 80% acetonitrile, and 0.1% formic acid) at a flow rate of 0.3 μL·min^−1^ by using the following gradients: 6% B for 5 min, 6–60% B for 125 min, 60–100% B for 5 min, 100% B for 5 min, 100–6% B for 2 min, and 6% B for 38 min.

The Orbitrap mass spectrometer was operated in a data‐dependent mode in which each full MS scan (60 000 resolving power) was followed by six MS/MS scans where the three most abundant molecular ions were dynamically selected and fragmented via collision‐induced dissociation by using a normalized collision energy of 35%, and the same three molecular ions were also scanned by HCD‐MS^2^ with collision energy of 45%. MS scans were acquired in profile mode and MS/MS scans in centroid mode. LTQ‐Orbitrap settings were as follows: spray voltage 2.0 kV, one microscan for MS^1^ scans at 60 000 resolution (fwhm at *m*/*z* 400), microscans for MS^2^ scans at 7500 resolution (fwhm at *m*/*z* 400); full MS mass range, *m*/*z* 400–1400; MS/MS mass range, *m*/*z* 100–2000. The ‘FT master scan preview mode’, ‘charge state screening’, ‘monoisotopic precursor selection’, and ‘charge state rejection’ were enabled so that only the 2+, 3+, and 4+ ions were selected and fragmented by collision‐induced dissociation and HCD.

#### 2.13.7 Database search and TMT quantification

Sequest, a tandem mass spectrometry data analysis program, was used for peptide matching and protein identification. The minimum number of peptides used for identification and quantification of proteins was one unique peptide. Sequest format files were generated by the proteome discoverer (v.1.2) data processing software (Thermo Scientific) by meeting the following criteria: database; enzyme, trypsin; maximum missed cleavages, 2; static modifications, carbamidomethylation (+57 Da), N‐terminal TMT 6‐plex (+229 Da), lysylTMT6‐plex (+229 Da); dynamic modifications, N‐terminal Cln‐pyro‐Glu (+17 Da), methionine oxidation (+16 Da), STY phosphorylation (+80 Da). MS peptide tolerance was set at 15 ppm; MS/MS tolerance at 0.05 Da. Peptides reported by the search engine were accepted only if they met the false discovery rate of *P* < 0.05 (target decoy database). For TMT quantification, the ratios of TMT reporter ion intensities in MS/MS spectra (up to six reporter ions ranging from *m*/*z* 126.12 to *m*/*z* 131.14) from raw data sets were used to calculate fold changes in proteins between control and case samples.

### 2.14 Proliferation assay

Cells were plated in a U‐shaped 96‐well plate at a density of 5 × 10^3^ cells/well. After the cells attached, they were treated with an increasing range of MJC13 drug concentrations for 1 h followed by the addition of R1881 (1 nm) for 24 h. Cell proliferation rates were then determined using CellTiter‐96 Nonradioactive cell proliferation assay (Promega) as per the manufacturer's instructions.

### 2.15 Animal studies

L(−)ID4 cells (2 × 10^6^) suspended in 100 μL of serum‐free RPMI 1640 medium containing matrigel (1 : 1 v/v; BD Biosciences, San Jose, CA, USA) were injected subcutaneously into the lower flanks of 4‐week‐old castrated (*C*) male C.B‐17 SCID mice (Taconic Biosciences, Rensselaer, NY, USA) using a 27‐gauge syringe. The C.B‐17 SCID mice were maintained at the Mercer University Vivarium. All studies were approved by the Clark Atlanta and Mercer University Committee for the use and care of animals.

#### 2.15.1 Preparation of tumor cells

L‐ID4 cells were grown in complete medium (10% v/v FBS in RPMI‐1640 medium). When cells were 70–80% confluent, 3–4 h before harvesting, medium was replaced with fresh medium to remove dead and detached cells. Then, fresh medium was removed, and cells were washed with PBS. After adding a minimum amount of trypsin/EDTA, cells were dispersed by adding complete medium (5 : 1) and then centrifuged immediately at 405 ***g*** for 5 min. After resuspending the cell pellet with complete medium (1 : 1), cells were counted using a hemocytometer.

#### 2.15.2 Tumor inoculation

The work area was prepared by disinfecting all hood surfaces with 70% ethanol. The inoculation area of each mouse was cleaned and sterilized with an alcohol pad. A freshly prepared cell suspension was agitated to prevent the cells from settling, and then mixed with matrigel. One hundred microliter of the mixture (containing 2 × 10^6^ L(−)ID4 cells) was injected subcutaneously into the lower flank of each of the 14 (4‐week‐old) castrated C.B‐17 SCID mice (Taconic Biosciences) using a 27‐gauge syringe. Tumor diameters were measured with digital calipers, and the tumor volume was calculated each week using the equation [V(volume) = (L(length) × W(width)^2^)/2]. At the end of the experiments, the mice were laid to rest by asphyxiation, the tumors were surgically removed and weighed, and the volume was measured. Harvested tumors were fixed in 10% buffered formalin. The fixed tumors were paraffin‐embedded, sectioned (5 μm), and either stained with hematoxylin and eosin (H&E) or used for immunohistochemistry (IHC). Images were captured using a Zeiss microscope (Zeiss, Thornwood, NY, USA) with an axiom cam version 4.5 (Zeiss, Thornwood, NY, USA) imaging system.

#### 2.15.3 MJC13 drug treatments

MJC13 drug therapy was started 5 weeks after inoculation, when the tumors reached an average volume of about 300 mm^3^. Mice were randomized into two groups with six mice in each group. The work area was prepared by disinfecting all hood surfaces with 70% ethanol. The tumor site of each mouse was cleaned and sterilized with an alcohol pad. The test group was administered 5 mg·kg^−1^ of MJC13 via intratumoral administration in the optimal co‐solvent formulation twice weekly for five consecutive weeks. The control group was administered the equivalent amount of co‐solvent vehicle without MJC13 following the same schedule. Tumor volumes were recorded prior to each treatment.

### 2.16 Serum PSA levels

ELISA was performed according to the manufacturer's instructions (Alpha Diagnostic International, San Antonio, TX, USA). At the end of the animal studies, blood was drawn from all the MJC13‐treated and/or untreated mice via cardiac bleed. After centrifuging to separate the serum fraction from the blood cells, the serum samples were analyzed for PSA levels using an enzyme‐linked immunosorbent assay. Briefly, the ELISA plates previously treated for the detection of human PSA by the manufacturer were used. Twenty‐five microliter of MJC13‐treated and/or untreated serum samples and standards were added to each well with 100 μL of AB–enzyme conjugate and incubated for 30 min at room temperature. The wells were washed with 300 μL of wash buffer followed by the addition of 100 μL TMB substrate per well and an incubation period of 15 min at room temperature. The reaction was stopped by adding 50 μL of stopping solution to all wells, and absorbance was measured at 450 nm using a Versa Max microplate spectrophotometer.

### 2.17 Data and statistical analysis

The NIH ImageJ (Schneider *et al*., 2012) was used for quantifying protein levels for the respective target proteins in the immunoblotting analysis. qRT‐PCR data were analyzed using the ∆∆C_*t*_ method (Sharma *et al*., 2012). The ChIP data were analyzed using % chromatin (1%) as input (Life Technologies, Carlsbad, CA, USA). Within group, Student's *t*‐test was used for evaluating the statistical differences between groups.

## 3 Results

### 3.1 ID4 interacts with AR through direct interaction with FKBP52 *in vitro*

**Figure 1:**
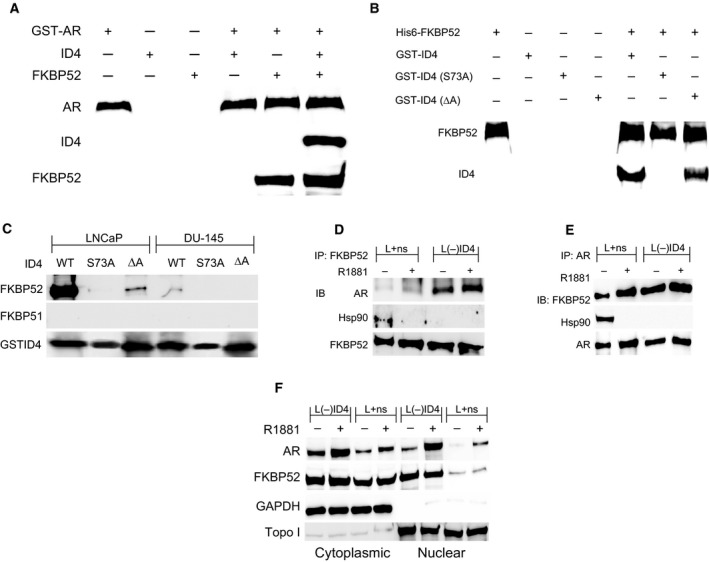
Effects of loss of ID4 on AR–FKBP52 interaction and nuclear translocation in L+ns and L(−)ID4 cells. (A) *In vitro* GST pull‐down assays were performed with purified, recombinant GST‐tagged AR, His6‐FKBP52, and recombinant ID4. Proteins were detected with respective primary antibodies to human AR, ID4, and FKBP52. (B) *In vitro* poly‐histidine pull‐down assays were performed with purified, recombinant His6‐tagged FKBP52, recombinant full‐length ID4, and truncated ID4 constructs ID4S73A (ID4 HLH mutant) and ID4∆A mutant (ID4 in which the alanine tract was deleted) as indicated. Proteins were detected with respective primary antibodies. (C) Pull‐down assays using LNCaP and DU‐145 whole‐cell lysates were performed with recombinant full‐length GST‐ID4, or truncated GST‐ID4 constructs ID4S73A (ID4 HLH mutant) and ID4∆A mutant. (D, E). The effects of loss of ID4 on AR–FKBP52 interaction and/or AR–Hsp90 complex dissociation were assessed in L+ns and L(−)ID4 cells by co‐immunoprecipitation and western blot analysis. Lysates prepared from cells grown in 10% charcoal‐stripped fetal bovine serum (csFBS) in the absence or presence of R1881 (10 nm) for 24 h were subjected to immunoprecipitation with either an antibody against FKBP52 (D), or AR (E) and immunoblotted for the indicated proteins. (F) Cytoplasmic versus nuclear immunolocalization of AR and FKBP52 in L+ns and L(−)ID4 cells in response to R1881 (10 nm). Topoisomerase (Topo1) and GAPDH were used to determine the purity and loading of nuclear and cytoplasmic extracts, respectively.

In order to address the mechanism of ID4‐mediated regulation of AR expression and activity (Patel *et al*., 2014; Sharma *et al*., 2013), we set out to identify ID4‐interacting proteins by co‐immunoprecipitation and subsequent mass spectrometry analysis using ID4‐specific antibody on the androgen‐sensitive prostate cancer LNCaP cells. Among different ID4 protein binding partners summarized in Table S1, we focused on FKBP52 that also acts as an AR co‐regulator/co‐chaperone. To confirm whether ID4 interacts with FKBP52 as part of a macromolecular complex involving AR, we performed an *in vitro* interaction assay using recombinant GST‐AR, ID4, and FKBP52. Recombinant human ID4 was passed through a glutathione‐coupled Sepharose column with bound GST‐AR or without 6xHis‐tagged FKBP52 in a cell‐free system. The bound proteins were eluted and detected on a western blot using ID4‐, FKBP52‐, and AR‐specific antibodies. As shown in Fig. 1A, ID4 was detected in elute only when 6xHis‐FKBP52 was passed through the column. These results suggested that ID4 does not directly bind to AR, but indirectly through FKBP52 that in turn is shown to bind AR (Fig. 1A). Direct interaction of ID4 with FKBP52 was next investigated using 6xHis‐tagged FKBP52 bound to a nickel column followed by incubation with recombinant ID4. As shown in Fig. 1B, a direct interaction between ID4 and FKBP52 in the absence of other proteins was observed. Importantly, mutation of the highly conserved amino acid (S73A) located within the ID4 HLH domain completely disrupted the interaction between ID4 and FKBP52, whereas the mutation in the poly‐alanine‐rich N‐terminal tract of ID4 (∆A mutant) significantly suppressed this interaction (Fig. 1B). These results were also recapitulated in GST‐ID4 pull‐down studies using total LNCaP and DU‐145 cell lysates, which confirmed that ID4 interacts with FKBP52 via its HLH domain (Fig. 1C). Given that ID4 is undetectable or weakly expressed in prostate cancer DU145 cells, they provide an ID4‐negative background as compared to the LNCaP cells wherein ID4 is endogenously expressed (Sharma *et al*., 2012), further confirming the domain‐specific interaction between ID4, FKBP52, and AR–protein complexes. These results led us to conclude that ID4 interacts with AR through FKBP52, which supports the co‐immunoprecipitation and mass spectrometry data shown in ST1.

### 3.2 ID4 knockdown promotes increased nuclear localization and co‐localization of AR and FKBP52 complexes

**Figure 2:**
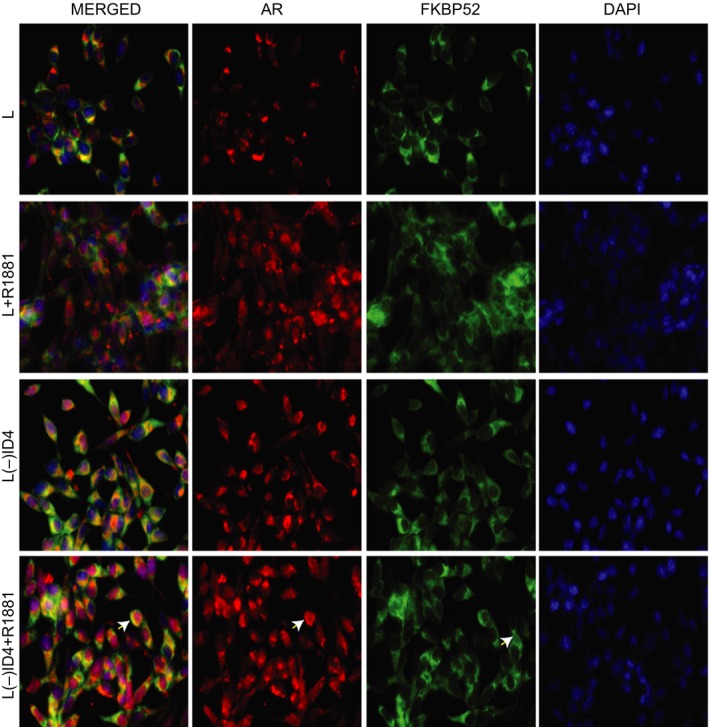
Co‐localization of AR and FKBP52 in L+ns and L(−)ID4 cell lines. Immunofluorescence of AR (red) and FKBP52 (green) in L+ns and L(−)ID4 cells in the absence or presence of R1881 (10 nm) for 24 h is shown. Red and green staining is protein specific and blue is nuclear (DAPI) (see respective insets). A representative image of three experiments is shown.

FKBP52 is an Hsp90‐associated co‐chaperone that has emerged as an attractive therapeutic target considering its functional specificity for a small subset of Hsp90 client proteins including androgen receptor (Guy *et al*., 2015). To test the effects of ID4 knockdown on AR–FKBP52 complex formation and/or AR–Hsp90 complex dissociation, we performed co‐immunoprecipitations of FKBP52 and AR in lysates of LNCaP (L+ns) and LNCaP‐ID4 (L(−)ID4) cells grown in the absence or presence of R1881 for 24 h. Western blot analysis of these immunoprecipitated samples demonstrated a significant increase in the protein–protein interaction between AR and FKBP52 (Fig. 1D,E). In addition, ID4 knockdown both in the absence or in the presence of R1881 also led to complex dissociation between AR and Hsp90, further implicating that cytoplasmic AR rapidly translocated to the nucleus for interaction with sequence‐specific androgen response elements. These results were also recapitulated in subcellular fractionation studies, which demonstrated that a large fraction of AR and FKBP52 is nuclear in L(−)ID4 than in L+ns cells (Fig. 1F). The effect of ID4 knockdown on AR and FKBP52 localization by immunocytochemistry (ICC) analysis also confirmed a significant increase in the nuclear localization and co‐localization (Fig. 2, white arrows) between AR and FKBP52 complexes in L(−)ID4 compared with those in control L+ns cells upon R1881 treatment.

### 3.3 Persistent AR and AR‐dependent expression in ID4‐knockdown LNCaP cells

**Figure 3:**
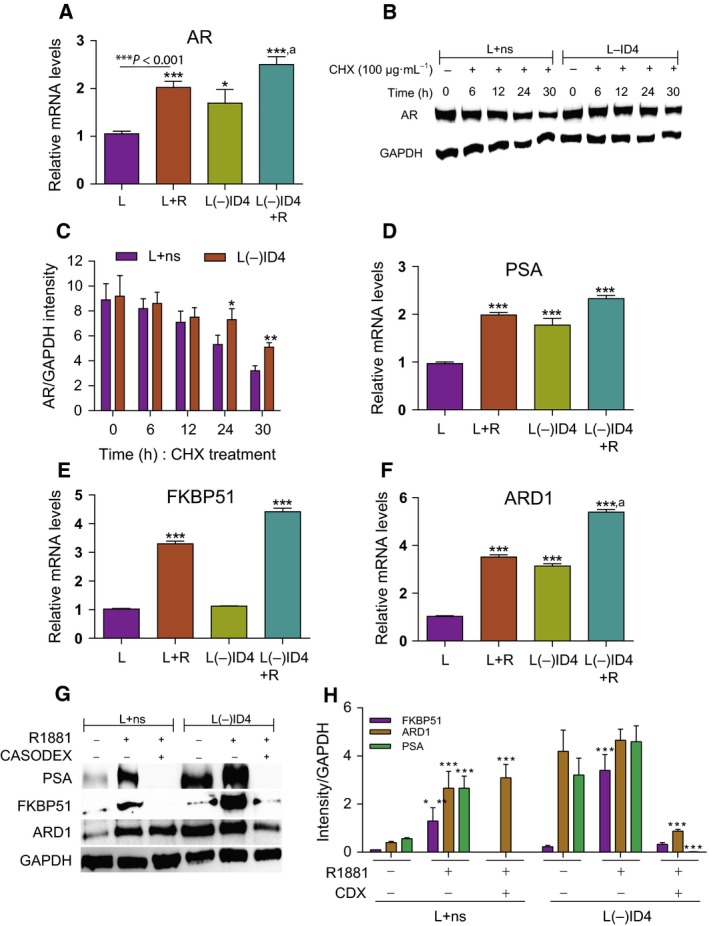
Expression of AR and AR‐regulated genes/proteins in L+ns and L(−)ID4 cell lines. (A) AR mRNA levels in L+ns and L(−)ID4 cells were determined by quantitative real‐time (qRT)‐PCR analysis in response to R1881 (R, 10 nm). Data (*n* = 3) are normalized to GAPDH followed by relative expression compared with the AR gene in L+ns (set to 1). (B) Immunoblot analysis of AR in response to cycloheximide (CHX, 100 μg·mL^−1^) treatment. Cells were treated with cycloheximide for the indicated time points (0, 6, 12, 24, and 30 h) followed by AR immunoblot analysis. (C) Semiquantitative AR protein levels (from B) normalized to GAPDH (loading control) and then to the individual AR protein levels in L+ns and L(−)ID4 cells. (D–F) qRT‐PCR analysis of AR‐regulated genes PSA, FKBP51, and ARD1 in L+ns and L(−)ID4 cells in response to R1881 (10 nm) in 10% csFBS. Data were normalized to GAPDH followed by relative expression compared with the respective genes in L+ns (set to 1). (G) AR‐dependent protein expression of PSA, FKBP51, and ARD1 in L+ns and L(−)ID4 cells cultured for 24 h in 10% csFBS before treatment with R1881 (10 nm) and/or R1881 (10 nm) ± Casodex (30 μm, antiandrogen) for another 24 h. (H) Semiquantitative FKBP51, ARD1, and PSA protein levels normalized to GAPDH (loading control) and then to the individual protein levels in L+ns and L(−)ID4 cells. Data are mean ± SEM (*n* = 3; *: *P* < 0.01, ***, ***^,a^: *P* < 0.001, where a is compared to L+R). Representative immunoblot data from three different experiments in triplicate are shown in the panel.

**Figure 4:**
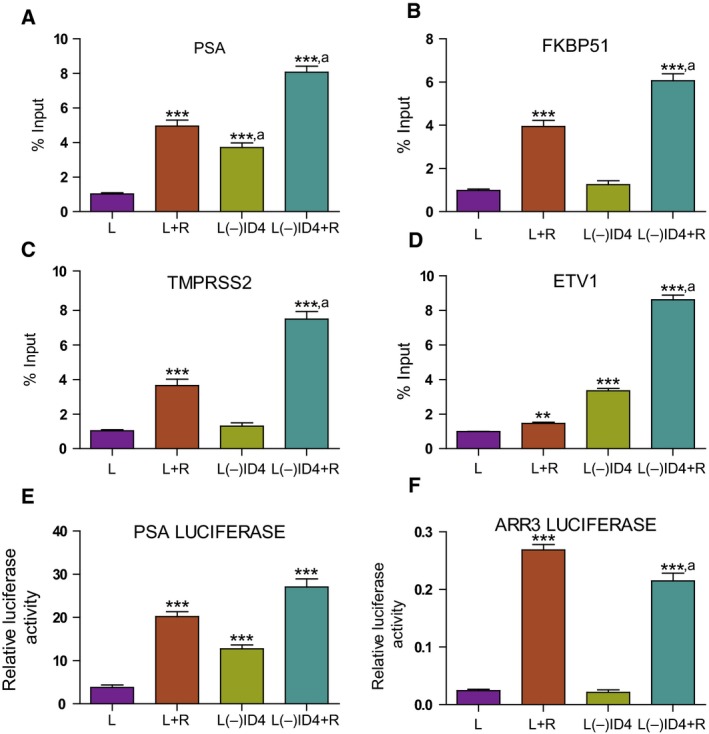
Effect of ID4 knockdown on AR‐mediated transactivation. (A–D) Chromatin immunoprecipitation (ChIP) assay demonstrating the enrichment of AR on the PSA, FKBP51, TMPRSS2, and ETV1 promoters. Androgen‐starved L+ns and L(−)ID4 cells were treated with R1881 (R, 10 nm) for 24 h. ChIP assay was then performed with AR‐specific antibodies. The data are expressed as percent input between L+ns and L(−)ID4 cells. (E, F) The AR transcriptional activity was determined by transiently transfecting L+ns and L(−)ID4 cells with the AR response element‐driven luciferase reporter plasmid (PSA luciferase), followed by treatment with R1881 (10 nm) or vehicle for 24 h. The data are normalized to Renilla luciferase. The luciferase reporter plasmid with mutated androgen response element (ARR3‐luciferase) was used as a negative control. The AR luciferase reporter activity in L(−)ID4 was normalized to L+ns cells. Data are mean ± SEM (*n* = 3; ***, ***^,a^: *P* < 0.001, where a is compared to L+R).

The primary role of AR in prostate cancer (PCa) is to regulate the expression of genes that are essential for prostate tumorigenesis (Lamont and Tindall, 2010). Increased AR‐dependent expression including *PSA*,* FKBP51*, and *ARD1* in prostate adenocarcinoma as compared to adjacent normal prostate (Fig. S1) was observed in The Cancer Genome Atlas (TCGA) prostate cancer adenocarcinoma (PRAD) gene expression (Illumina Hiseq) database. In Fig. 3A, real‐time qPCR results demonstrated a significantly higher constitutive AR expression which increased further in response to androgens in L(−)ID4 compared with that in L+ns cells. Given that AR protein stability has been found to be increasingly associated with nuclear shuttling and elevated AR levels, we examined the effect of ID4 knockdown on AR protein turnover rates with the cycloheximide chase assay. As shown in Fig. 3B,C, cycloheximide treatment delayed AR protein degradation rate at 24 and 30 h in L(−)ID4 cells compared with those in L+ns cells. These data suggest that loss of ID4 leads to an increase in the stability of AR protein levels (*P* < 0.001). Also, AR‐dependent expression including PSA, FKBP51, and ARD1 was found to be significantly altered in L(−)ID4 cells compared with those in L+ns cells, both at the transcript (Fig. 3D–F) and at the protein levels (Fig. 3G,H) in an androgen‐dependent (FKBP51) or androgen‐independent manner (PSA, ARD1). In both these cell lines, AR‐dependent expression including PSA, FKBP51, and ARD1 was reversed by the antiandrogen Casodex, suggesting androgen‐dependent AR regulation. Furthermore, qPCR analysis on chromatin‐immunoprecipitated (ChIP) DNA with AR antibody demonstrated a significant increase in the binding of AR to its respective response elements on PSA, FKBP51, TMPRSS2, and ETV1 promoters in L(−)ID4 compared with those in L+ns cells (Fig. 4A–D). In a functional transcriptional assay using a AR response element (PSA luciferase) reporter plasmid, the relative PSA luciferase activity increased significantly in L(−)ID4 compared with those in control L+ns cells, both in the absence or in the presence of R1881 (Fig. 4E,F) (*P* < 0.001). Together, these results confirmed that knockdown of ID4 in LNCaP cells stabilizes AR protein levels, potentiates AR‐dependent expression and activity, further implicating that ID4 functions as a selective regulator of AR activity.

### 3.4 Knockdown of ID4 promotes androgen receptor‐mediated transcriptional activation through Hsp27 and FKBP52

**Figure 5:**
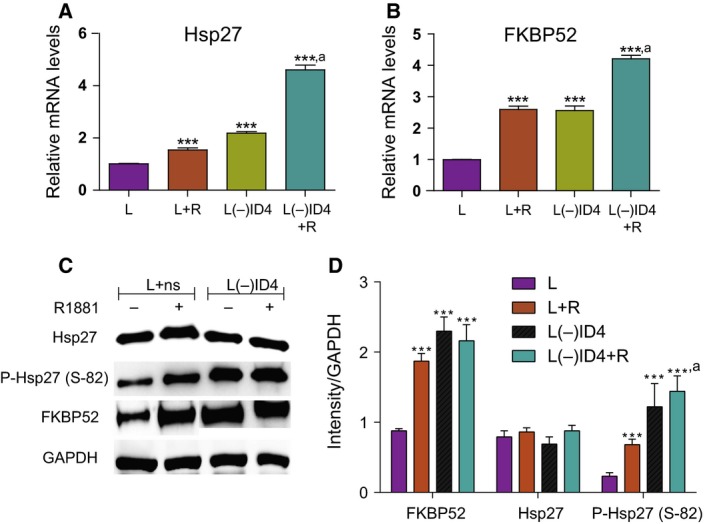
Hsp27 and FKBP52 expression in L+ns and L(−)ID4 cell lines. (A, B) Comparison of AR co‐chaperones Hsp27 (A) and FKBP52 (B) mRNA levels in L+ns and L(−)ID4 cells by qRT‐PCR analysis. Cells were treated for 24 h in the absence or presence of R1881 (10 nm) in 10% csFBS. Data were normalized to GAPDH followed by relative expression compared with the respective genes in L+ns (set to 1). (C) Protein levels of AR co‐chaperones Hsp27, phosphorylated (p) Hsp27 [Ser82], and FKBP52 in L+ns and L(−)ID4 cells. (D) Semiquantitative FKBP52, Hsp27, and P‐Hsp27 (S‐82) protein levels normalized to GAPDH and then to the individual protein levels in L+ns and L(−)ID4 cells. Data are mean ± SEM (*n* = 3; ***, ***^,a^: *P* < 0.001, where a is compared to L+R). Representative data from three different experiments in triplicate are shown in the panel.

**Figure 6:**
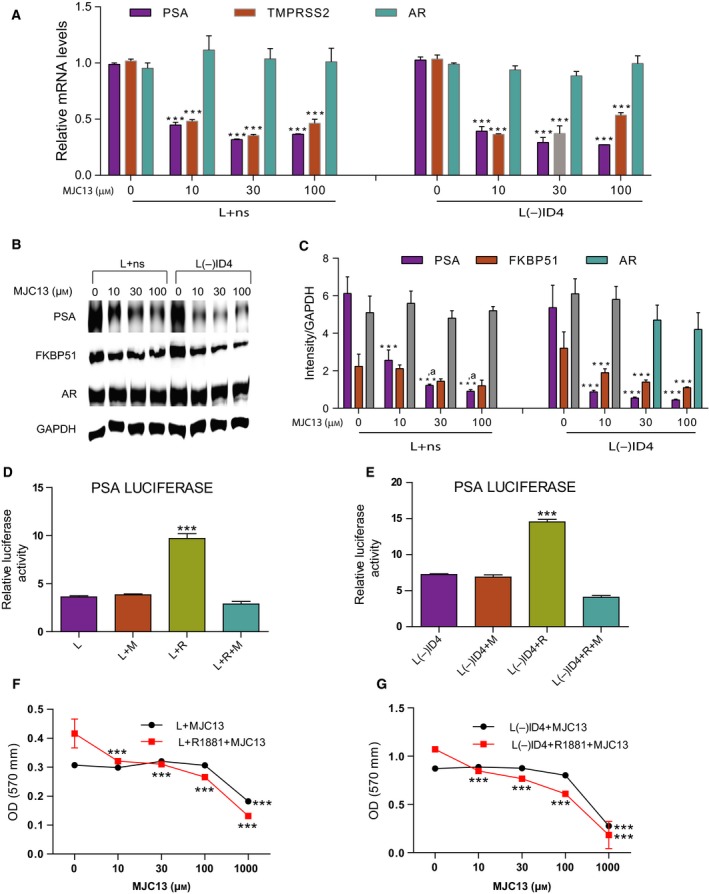
Inactivation of ID4 in LNCaP cells promotes constitutive AR activation through FKBP52. (A) PSA, TMPRSS2, and AR gene expression levels in L+ns and L(−)ID4 cells were assessed by qRT‐PCR analysis. Cells were treated for 24 h with increasing concentrations of MJC13 as indicated above in the presence of 10% FBS. Data were normalized to GAPDH followed by relative expression compared with the respective genes in L+ns and L(−)ID4 (set to 1). (B) Immunoblot analysis of AR and AR‐regulated proteins PSA and FKBP51 in L+ns and L(−)ID4 cells treated for 24 h with increasing concentrations of MJC13 as indicated above. (C) Semiquantitative protein expression (from B) of PSA, FKBP51, and AR protein levels was normalized to GAPDH and then to the individual protein levels in L+ns and L(−)ID4 cells. (D, E) The AR transcriptional activity was determined by transiently transfecting L+ns and L(−)ID4 cells with the AR response element‐driven luciferase reporter plasmid (PSA luciferase), then treated with MJC13 (30 μm) for 1 h followed by the addition of R1881 (1 nm) or vehicle for additional 24 h. The data are normalized to Renilla luciferase. The mutated AR luciferase reporter plasmid (ARR3‐luciferase) was used as a negative control. The AR luciferase reporter activity in L+ns and L(−)ID4 cells treated with MJC13 was normalized to control L+ns and L(−)ID4 cells. (F, G) Proliferation rate of L+ns and L(−)ID4 cells treated with MJC13 concentrations as indicated above in the absence or presence of R1881 (1 nm) for 24 h. Data are presented as mean ± SEM (*n* = 3; ***, ***^,a^: *P* < 0.001, where a is compared to L+R). Representative data from three different experiments in triplicate are shown.

In order to better understand the underlying molecular mechanism through which loss of ID4 potentiates AR transcriptional activity, proteomic analysis was performed to analyze differential protein expression in L(−)ID4 cells compared with that in L+ns cells. Among the most notable ones, there were 46 proteins up‐regulated and 34 proteins down‐regulated in L(−)ID4 relative to L+ns cell lines (Tables S2 and S3). More importantly, these results demonstrated a 6.8‐fold increase in heat shock protein 27 (Hsp27) protein levels, whereas a 3.4‐fold increase in FK506‐binding protein 4 (FKBP52) protein levels following the loss of ID4 in LNCaP cells. These two target proteins are well‐established molecular chaperone and co‐chaperone proteins, respectively (De Leon *et al*., 2011; Rocchi *et al*., 2004; Storer Samaniego *et al*., 2015; Zoubeidi *et al*., 2007), and are known to be involved in modulating AR signaling during prostate cancer progression and development. Moreover, real‐time PCR and immunoblotting results confirmed a significant up‐regulation of Hsp27 and FKBP52 expression in L(−)ID4 cells compared with those in L+ns cells, both at the transcript (Fig. 5A,B) and at the protein levels (Fig. 5C,D), further supporting the current quantitative proteomic dataset. Interestingly, Hsp27 phosphorylation levels on Ser^82^ residue were found to be significantly elevated in the ID4‐knockdown LNCaP cells (Fig. 5C,D). Previous studies have shown that androgen‐induced phosphoactivation of Hsp27 on Ser^78^ and Ser^82^ residues displaces Hsp90 from a complex with AR to chaperone AR into the nucleus (Zoubeidi *et al*., 2007). Consistent with previous studies, ICC results demonstrated increased nuclear co‐localization between AR and P‐Hsp27 protein complexes in L(−)ID4 compared with those in control L+ns cells (Fig. S2, white arrows). FKBP52 has been characterized as an important regulator of AR‐mediated transcription (De Leon *et al*., 2011; Guy *et al*., 2015; Storer Samaniego *et al*., 2015). Collective observations prompted us to further investigate whether knockdown of ID4 promotes AR activation through FKBP52. The effects of MJC13 treatment (inhibitor of FKBP52‐regulated AR activity) (De Leon *et al*., 2011; Liang *et al*., 2016; Storer Samaniego *et al*., 2015) on AR and AR‐dependent expression were assessed in L+ns and L(−)ID4 cells. Real‐time PCR and immunoblot analyses demonstrated that MJC13 effectively inhibited endogenous PSA, TMPRSS2, and FKBP51 expression in a dose‐dependent manner (Fig. 6A–C). However, the inhibitory effects of MJC13 had no impact on AR mRNA and protein levels. A similar decrease in the relative PSA luciferase activity was observed between L+ns and L‐ID4 cells after treatment with MJC13 (Fig. 6D,E) (*P* < 0.001). In addition, MJC13 also inhibited androgen‐dependent cell proliferation in L+ns and L(−)ID4 cells at concentrations consistent with those observed to be effective in reporter assays (Fig. 6F,G). Together, these results indicated that with subsequent loss of ID4, Hsp27 and FKBP52 promote nuclear translocation and transcriptional activity of AR, followed by increased cell proliferation in prostate cancer L(−)ID4 cell lines.

### 3.5 Inhibition of FKBP52‐regulated AR activity attenuates tumor growth of subcutaneous xenografts *in vivo*

**Figure 7:**
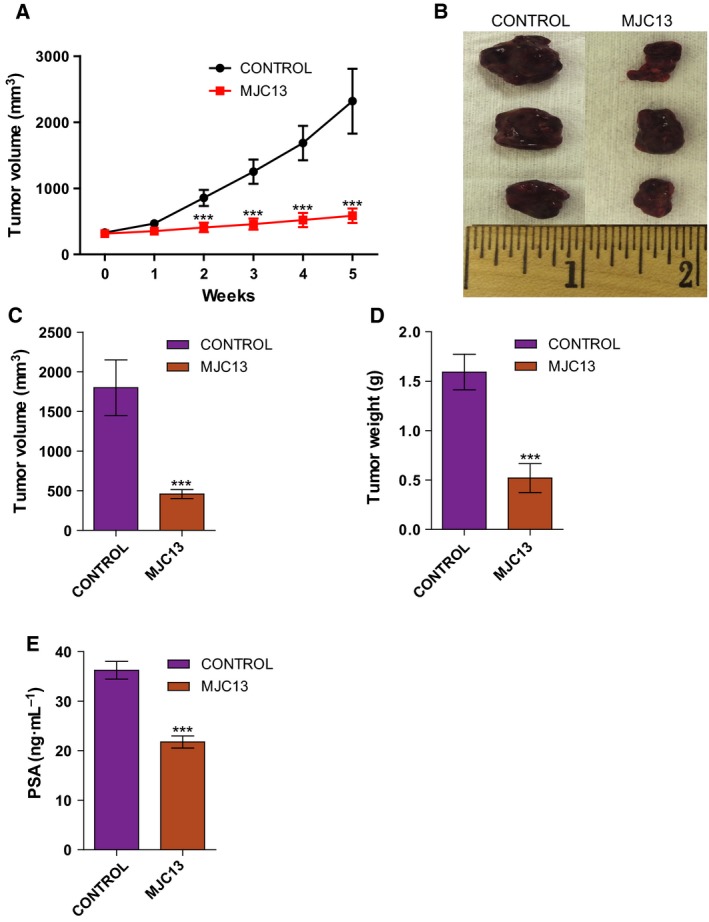
Effects of MJC13 treatment on tumor growth *in vivo*. (A) Volumes of the MJC13‐treated and/or untreated tumors were measured weekly (expressed as mm^3^). Vehicle or MJC13 was administered via intratumoral injection twice weekly for a total of five consecutive weeks. The castrated male SCID mice were later sacrificed after five consecutive weeks of vehicle or MJC13 treatments. (B) Representative xenograft images of respective tumors untreated/treated with MJC13 are shown. (C, D) Respective volumes and weights of the L(−)ID4 tumors after excision from the mice untreated/treated with MJC13. (E) Blood samples were collected from MJC13‐treated and/or untreated xenograft models via cardiac bleed, serum fraction was isolated, and then serum PSA levels (ng PSA per ml of serum) were determined by ELISA. Data are presented as mean ± SEM (*n* = 6; ***: *P* < 0.001, where *n* = 6 animals in each of the MJC13‐treated and untreated groups for the data shown in the panel).

To further validate the *in vitro* cell line data procured so far, LNCaP‐ID4 cells were subcutaneously injected into the flanks of 4‐week‐old previously castrated male SCID mice to investigate the effect of inhibiting FKBP52‐AR signaling on the tumor growth via MJC13 drug treatments. Therapy was started 5 weeks after inoculation, when the tumors reached an average volume of about 300 mm^3^. Throughout the entire course of intratumoral drug treatments, the average tumor volume versus time for control (vehicle‐treated) and test (MJC13‐treated) groups was also determined for a total of five consecutive weeks. The data demonstrate that tumor growth was significantly inhibited starting at week 2 after MJC13 treatment (Fig. 7A). At the end of the experiment (after five consecutive weeks of vehicle or MJC13 drug treatments), the tumors were excised and volume and weights were measured. In the treatment group, MJC13 drug treatments significantly attenuated the tumor growth of subcutaneous xenografts *in vivo* (75%) as compared to the control (vehicle‐treated) tumors in the mice (Fig. 7B). Also, the overall tumor weights (67.35%) and volumes (75%) were found to be significantly attenuated in the MJC13‐treated xenografts as compared to the control model of the castrated mice (Fig. 7C,D). In addition, ELISA results of serum PSA levels from MJC13‐treated and/or untreated xenograft models demonstrated that MJC13 effectively inhibited PSA secretion (Fig. 7E), one of the major AR‐dependent target genes. These results are consistent with the previously shown cell line data (Fig. 6), further confirming that inhibition of FKBP52‐regulated AR activity significantly attenuates AR signaling in L(−)ID4 cells, both *in vivo* and *in vitro*. Collectively, these results indicated that knockdown of ID4 enhances tumorigenicity of prostate cancer cells in the CRPC conditions, more importantly through FKBP52‐mediated AR signaling.

### 3.6 Xenograft morphology and ID4, AR, PSA, FKBP1, FKBP52, KI67, and lamin expression

**Figure 8:**
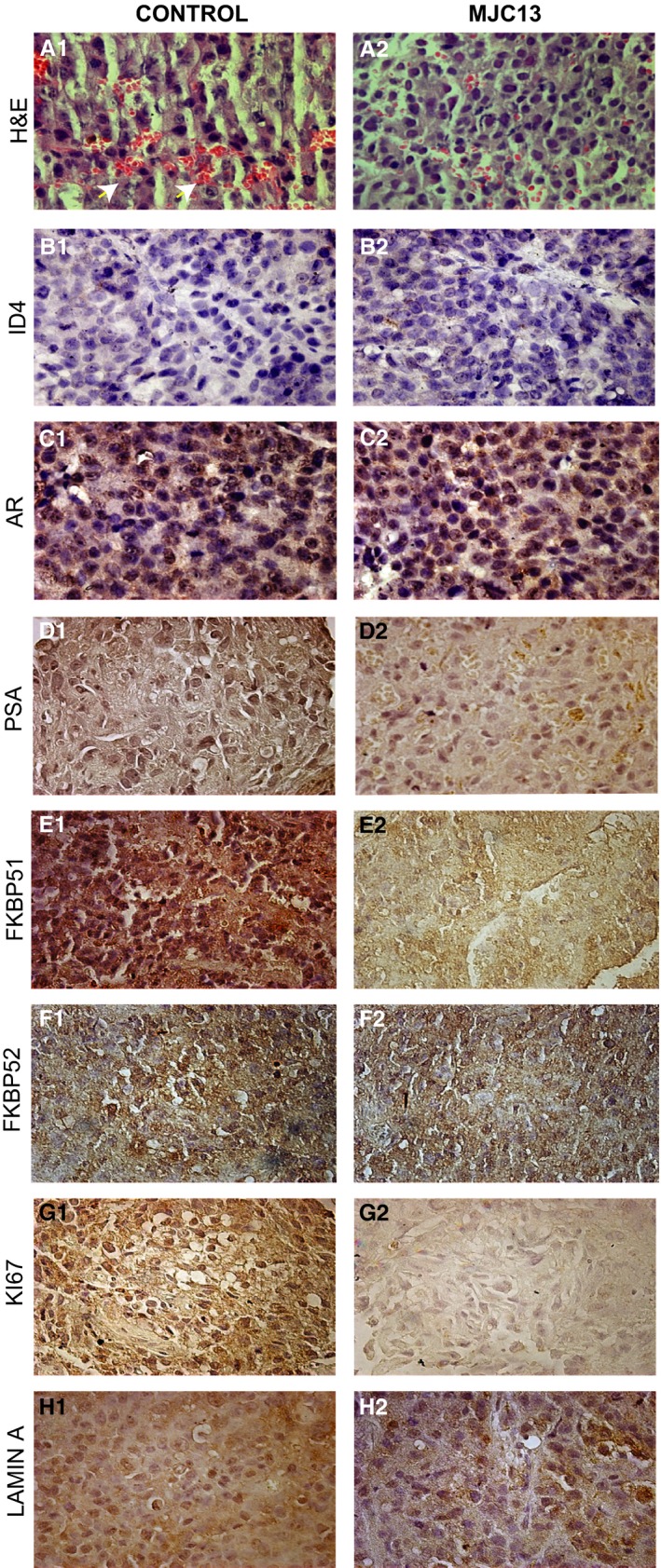
Histological and immunohistological analyses of L(−)ID4 xenografts treated and/or untreated with MJC13 from castrated SCID mice. (A1, A2) Hematoxylin and eosin staining of tumor xenografts. (B1–H2) Immunohistochemical localization (brown staining) of ID4 (B1, B2), AR (C1, C2), PSA (D1, D2), FKBP51 (E1, E2), FKBP52 (F1, F2), KI67 (G1, G2), and human‐specific lamin A (H1, H2) in tumor xenografts. All images are representative and at 400× magnification.

The histological examination of the tumors demonstrated an abundance of infiltrating red blood cells in the control as compared to the MJC13‐treated L(−)ID4 xenografts, suggesting decreased vascularization in MJC13‐treated tumors (Fig. 8A1,A2, white arrows). As expected, ID4 immunoreactivity was undetectable in L(−)ID4 xenografts (Fig. 8B1,B2). IHC analysis of the xenograft tumors showed no change in AR and FKBP52 expression levels in both MJC13‐treated and untreated xenograft tumors (Fig. 8C1,C2,F1,F2). However, the AR‐dependent expression including PSA and FKBP51 was found to be significantly attenuated in the MJC13‐treated group than in control L(−)ID4 xenografts (Fig. 8D1–E2). Concomitant with the decreased serum PSA levels in the MJC13‐treated xenografts (Fig. 7E), these results further confirmed that MJC13 effectively inhibited FKBP52‐mediated AR signaling *in vivo*. In addition, MJC13 also reduced the expression level of KI67 as compared to the control group (Fig. 8G1,G2). Thus, increased tumor growth in the control group resulted from an increase in proliferation of L(−)ID4 cells *in vivo*. Predominant human‐specific lamin A expression observed in both MJC13‐treated and untreated L(−)ID4 xenografts (Fig. 8H1,H2) further suggested that increased tumor volume was due to the expansion of LNCaP cells and not due to mouse‐derived stromal cells. Overall, these results indicated that MJC13 blocks AR‐dependent expression and proliferation in the L(−)ID4 xenografts.

## 4 Discussion

Studies have previously shown that epigenetic silencing of ID4 due to promoter hypermethylation appears to be the key mechanism in many cancers including prostate (Carey *et al*., 2009; Chinaranagari *et al*., 2014; Sharma *et al*., 2012). In particular, a strong association between ID4 and AR‐dependent expression including PSA, FKBP51, and ARD1 in hormone‐refractory metastatic PCa shows direct clinical relevance of a possible cross‐talk between ID4 and AR. The present study demonstrated that ID4 selectively regulates AR activity through direct interaction with FKBP52. Given that ID4 expression inversely correlates with CRPC compared with that in hormone‐naïve prostate cancer (Patel *et al*., 2014), we primarily focused our attention on the LNCaP and stable LNCaP(−)ID4 cell lines, which mimics decreased ID4 expression in PCa (Knowell *et al*., 2013; Patel *et al*., 2014). In addition, these two cell lines closely resemble the androgen sensitivity of androgen receptor, in the absence or presence of ID4 and its subsequent transition to a castration‐resistant environment *in vitro* and *in vivo* (Igawa *et al*., 2002; Karan *et al*., 2002; Patel *et al*., 2014).

Co‐immunoprecipitation followed by mass spectrometry analysis identified different ID4 binding partners; most of these proteins and their biological significance have been well documented. Intriguingly, our list of ID4 putative interactors includes a remarkable number of molecular chaperones and co‐chaperones, a wide gene family whose components are involved in processes of protein folding, activation, trafficking, and transcriptional activity of most steroid receptors, including AR (Zoubeidi *et al*., 2007). For example, Hsp90 is an important molecular chaperone, and the relationship between the chaperone functioning of Hsp90 with AR stability, conformation, and modulation of ligand binding is well characterized (He *et al*., 2013; Smith and Toft, 2008). However, Hsp10 is a 10‐kDa highly conserved, mitochondrion‐resident protein, which co‐chaperones with another mitochondrial heat shock protein Hsp60 for protein folding as well as the assembly and disassembly of important protein complexes (Jia *et al*., 2011). In addition to these ID4 protein partners, the current approach also identified FKBP52 as a potential ID4 binding partner in prostate cancer LNCaP cells. Furthermore, mutation of the conserved serine to alanine (ID4S73A HLH mutant) in the HLH domain of ID4 resulted in loss of its interaction with FKBP52, suggesting that the interaction with FKBP52 is dependent on the intact HLH domain of ID4. Interestingly, deletion of the alanine residues (39–48, ID4∆ mutant) in ID4 (specific only to ID4 and no other ID family members) did not result in complete abrogation of the binding, but the interaction appeared to be significantly weaker as compared to wild‐type ID4. Collectively, these results suggest the functional significance of HLH domain and poly‐alanine stretch of ID4 in ID4–FKBP52 interactions. In this study, we did not observe a direct interaction between ID4 and AR, further implicating that ID4 could possibly regulate AR activity indirectly through FKBP52. Furthermore, knockdown of ID4 in LNCaP cells resulted in increased nuclear localization and co‐localization of AR–FKBP52 protein complexes, following the androgen‐induced AR–Hsp90 complex dissociation.

AR remains important in the development and progression of prostate cancer, and the majority of androgen‐independent or hormone‐refractory prostate cancers express AR (Heinlein and Chang, 2004), which in part is associated with the extensive re‐programming of its transcriptional activity (Pomerantz *et al*., 2015). Oncogenic activation of AR during the development and progression of PCa, particularly in the early to late stages of CRPC, is largely dependent on multiple factors including increased protein stability, post‐translational modifications, interactions with specific co‐regulators, and ligand specificity. Following the knockdown of ID4 in LNCaP cells [L(−)ID4], increased protein stability along with the constitutive activation of androgen receptor marked by increased AR‐dependent expression including PSA, FKBP51, and ARD1 as well as ability to interact with the androgen response elements of the respective androgen‐responsive genes such as PSA, FKBP51, TMPRSS2, and ETV1 unequivocally supports the tumor suppressor role of ID4 in the regulation of AR expression and activity. In the current study, we also observed a significant increase in the sensitivity of androgen receptor response to R1881 treatment in L(−)ID4 cells, further implicating the potential role of FKBP52 in modulating the sensitivity of AR response to androgens in the absence of ID4.

In recent years, Hsp27 and FKBP52 have been identified among the most consistently overexpressed genes in hormone‐refractory prostate cancer xenografts (Liang *et al*., 2016; Rocchi *et al*., 2004). Quantitative proteomic analysis identified distinct protein signatures in L(−)ID4 cells including important AR co‐chaperones such as Hsp27 and FKBP52, the two well‐characterized nuclear transporters (Zoubeidi *et al*., 2007) and transcriptional activators (De Leon *et al*., 2011; Storer Samaniego *et al*., 2015; Storer *et al*., 2011; Yong *et al*., 2007) of androgen receptor. In comparison with elevated Hsp27 mRNA levels, we did not observe any difference in the total Hsp27 protein levels in L+ns and L(−)ID4 cells, possibly due to technical limitations of the western blotting analysis to distinguish relatively smaller quantitative changes in the protein levels. However, quantitative mass spectrometry studies were able to distinguish changes between total Hsp27 protein levels in these two cell lines. Thus, it is anticipated that similar results can also be replicated via immunoblotting analysis by loading relatively smaller amounts of respective protein samples (5 μg). Furthermore, the protein levels of the phosphoactivated form of Hsp27 on Ser^82^ residue were found to be significantly increased following the loss of ID4 in L+ns cells. Increased cellular localization and co‐localization between AR and P‐Hsp27 protein complexes further highlights the pivotal role of Hsp27 in the nuclear transport of AR in L(−)ID4 cells. Furthermore, inhibiting FKBP52‐regulated AR activity via MJC13 significantly inhibited AR‐dependent expression, activity, and androgen‐stimulated proliferation in L(−)ID4 cells. Collectively, these results demonstrate that with ID4 knockdown [L(−)ID4], Hsp27‐dependent nuclear translocation and FKBP52‐potentiated AR signaling further lead to increased tumorigenicity, both *in vitro* and *in vivo*. However, in the present study, an important question that remains to be addressed is the underlying molecular mechanism through which loss of ID4 promotes AR, FKBP52, and Hsp27 expression. It is quite possible that ID4 interaction/cross‐talk with different transcription factors/signaling pathways may contribute to the regulation of these genes.

Given that L+ns cells form relatively smaller‐sized tumors in the castration‐resistant environment, we primarily focused our *in vivo* studies using xenograft mice model on the stable L(−)ID4 cells, which resemble CRPC phenotype in the absence of ID4 (Patel *et al*., 2014). Consistent with our previous observation, in the present study loss of ID4 enhanced *in vivo* tumor growth in the castration‐resistant environment, more importantly through FKBP52‐mediated AR signaling pathway. Consequently, inhibiting FKBP52‐regulated AR activity through MJC13 drug treatment in L(−)ID4 xenografts significantly attenuated the tumor growth *in vivo*. Concomitant with xenograft studies, molecular techniques including ELISA and IHC analysis highlight the importance of AR signaling in the tumor growth in an androgen‐depleted environment, further implicating the specificity of tumor‐suppressive effects of ID4 in the selective regulation of AR activity in PCa.

In summary, the data presented in the current study combined with those in our previous publications, an ID4‐regulated AR signaling model is depicted in Fig. S3 In this pathway, ID4 appears to selectively regulate AR activity through direct interaction with FKBP52 *in vitro*. Our data also suggest that in the absence of ID4, FKBP52 significantly potentiated AR signaling leading to increased proliferation and tumor growth. Regardless of the manner in which ID4 and FKBP52 interact to regulate AR activity, our data suggest a clear inhibitory relationship between these two target proteins. Furthermore, to validate the functional relationship between ID4 and FKBP52 in regulating AR signaling, *in vitro* pull‐down studies using multiple domain‐specific ID4 and FKBP52 constructs need to be performed. Given the critical role of the FKBP52 proline‐rich loop that overhangs the PPIase pocket in the regulation of AR activity (Storer Samaniego *et al*., 2015), we speculate that ID4 could target distinct regulatory sites within FKBP52 for the disruption of AR signaling in PCa. Therefore, our data suggest that regulating AR activity indirectly through FKBP52 either by ID4‐ or by FKBP52‐specific inhibitors such as MJC13 would be a valuable pharmacological tool for selectively attenuating persistent AR activity in CRPC, irrespective of hormonal milieu of the cancer.

## Author contributions

JBJ and JC conceived and designed the project. JBJ, DP, DJM, PS, JZ, DHB, YG, PN, SKK, RP, and GW acquired the data. JCS, HX, and MC supplied the necessary reagents with reference to MJC13‐related studies. JBJ and JC analyzed and interpreted the data. JBJ wrote the first draft, and JC edited the paper.

## Supporting information


**Fig. S1.** Expression of ID4 correlates inversely with major AR‐regulated expression in prostate cancer.

**Fig. S2.** Co‐localization of AR and P‐Hsp27 in L+ns and L(−)ID4 cell lines.

**Fig. S3.** Schematic model of ID4 regulated AR activity in prostate cancer cells.

**Table S1.** ID4 binding partners identified from prostate cancer LNCaP cells using a two‐step co‐immunoprecipitaton and mass spectrometry approach.

**Table S2.** List of significantly up‐regulated proteins in L(−)ID4 compared with those in L+ns cells.

**Table S3.** List of significantly down‐regulated proteins in L(−)ID4 compared with those in L+ns cells.

**Appendix S1.** Antibodies and reagents for Immunoblots, co‐immunoprecipitation, Immunocytochemistry, Immunohistochemistry, and ChIP assays.
**Appendix S2.** List of real‐time PCR primer sequences.
**Appendix S3.** List of quantitative RT‐PCR primer sequences used for ChIP analysis.
