# Supplementary material for: Inactivation of ID4 promotes a CRPC phenotype with constitutive AR activation through FKBP52
Source: Mol Oncol. 2017 Mar 2;11(4):337–57. doi: 10.1002/1878-0261.12028 (PMC5378613; doi:10.1002/1878-0261.12028)
Supplement: Supplementary file 4 — Table S1. ID4 binding partners identified from prostate cancer LNCaP cells using a two‐step co‐immunoprecipitaton and mass spectrometry approach. [file MOL2-11-337-s004.docx]

| **Protein Name** | **Symbol** | **Accession #** | **Gene ID** | **Coverage** | **Score** |
| --- | --- | --- | --- | --- | --- |
| Inhibitor of DNA binding 4 | ID4 | IPI:IPI00026864.1 | 3400 | 18.01 | 1503.05 |
| PYD and CARD domain containing | PYCARD | IPI00221362.3 | 29108 | 10.37 | 165.65 |
| Nucleolin | NCL | IPI00444262.3 | 4691 | 4.64 | 153.89 |
| 49 kDa uncharacterized protein | ------- | IPI:IPI00880053.1 | ---- | 3.79 | 143.33 |
| Complement component 1, q subcomponent binding protein | C1QBP | IPI:IPI00014230.1 | 708 | 4.96 | 140.1 |
| ATP synthase, H+ transporting, mitochondrial F1 complex, beta polypeptide | ATP5B | IPI00792534.1 | 506 | 20 | 139.1 |
| Annexin A2 | ANXA2 | IPI00903334.1 | 302 | 18.23 | 137.7 |
| Parkinson protein 7 | DJ-1 | IPI:IPI00298547.3 | 11315 | 10.05 | 124.04 |
| Protein disulfide isomerase family A, member 6 | PDIA6 | IPI:IPI00644989.2 | 10130 | 11.14 | 118.06 |
| Lactate dehydrogenase A | LDH1 | IPI00795075.1 | 3939 | 6.22 | 105.96 |
| 22 kDa uncharacterized protein | ------- | IPI00797149.2 | ---- | 6.67 | 100.16 |
| Transketolase | TKT | IPI00946864.1 | 7086 | 5.09 | 94.18 |
| SERPINE1 mRNA binding protein 1 | SERBP1 | IPI:IPI00412714.3 | 26135 | 4.13 | 88.19 |
| Tyrosine 3-monooxygenase activation protein, theta | YWHAQ | IPI:IPI00018146.1 | 10971 | 7.76 | 87.47 |
| Tyrosine 3-monooxygenase activation protein, beta | YWHAB | IPI00759832.1 | 7529 | 7.79 | 79 |
| Putative uncharacterized protein MCM6 | ------- | IPI00908729.1 | ---- | 20.48 | 78.4 |
| Lymphocyte cytosolic protein 1 (L-plastin) | LCP1 | IPI:IPI00643270.1 | 3936 | 28.79 | 76.2 |
| 7 kDa uncharacterized protein | ------- | IPI00793702.1 | ---- | 40.35 | 74.46 |
| Translocase of outer mitochondrial membrane 70 homolog A | TOMM70A | IPI00793189.2 | 9868 | 7.73 | 72.1 |
| Protein disulfide isomerase family A, member 3 | PDIA3 | IPI00893541.1 | 2923 | 11.38 | 65.19 |
| Hypoxia up-regulated 1 | HYOU1 | IPI00922127.1 | 10525 | 2.33 | 64.55 |
| Heat shock 70kDa protein 2 | HSP70-2 | IPI00902596.1 | 3306 | 2.42 | 62.56 |
| RAN binding protein 1 | RANBP1 | IPI00878440.1 | 5902 | 6.63 | 61.73 |
| Canopy FGF signaling regulator 2 | CNPY2 | IPI:IPI00443909.1 | 10330 | 8.24 | 59.01 |
| GTPase activating protein (SH3 domain) binding protein 1 | G3BP1 | IPI:IPI00012442.1 | 10146 | 3.86 | 57.6 |
| FK506 binding protein 4, 59kDa | FKBP52 | IPI:IPI00219005.3 | 2288 | 3.7 | 56.53 |
| 22 kDa uncharacterized protein | ------- | IPI00796633.1 | ---- | 7.14 | 55.82 |
| Heterogeneous nuclear ribonucleoprotein U (scaffold attachment factor A) | HNRNPU | IPI:IPI00963858.1 | 3192 | 4.13 | 53.21 |
| Heat shock protein 90kDa alpha (cytosolic) | HSP90AA1 | IPI00604607.2 | 3320 | 3.7 | 50.3 |
| Heat shock 10kDa protein 1 | HSPE1 | IPI00916345.1 | 3336 | 25.53 | 50.11 |
