# Supplementary material for: Inactivation of ID4 promotes a CRPC phenotype with constitutive AR activation through FKBP52
Source: Mol Oncol. 2017 Mar 2;11(4):337–57. doi: 10.1002/1878-0261.12028 (PMC5378613; doi:10.1002/1878-0261.12028)
Supplement: Supplementary file 5 — Table S2. List of significantly up‐regulated proteins in L(−)ID4 compared with those in L+ns cells. [file MOL2-11-337-s005.docx]

| **Protein Name** | **Symbol** | **Accession #** | **Gene ID** | **Coverage** | **Fold Change** |
| --- | --- | --- | --- | --- | --- |
| Heat shock protein 27 | HSP27 | IPI00025512.2 | 3315 | 25.85 | 6.836 |
| Aldehyde dehydrogenase 1 | ALDH1A1 | IPI00910463.1 | 216 | 2.31 | 4.836 |
| Sorbitol dehydrogenase | SORD | IPI00216057.6 | 6652 | 11.76 | 4.347 |
| Membrane metallo-endopeptidase | MME | IPI00247063.3 | 4311 | 1.47 | 4.293 |
| P antigen family, member 1 [prostate associated] | PAGE1 | IPI00025748.2 | 8712 | 7.53 | 3.816 |
| Epoxide hydrolase 1 | EPHX1 | IPI00009896.1 | 2052 | 9.45 | 3.732 |
| Nucleobindin-2 | NUCB2 | IPI00009123.2 | 4925 | 2.14 | 3.582 |
| FK506 binding protein 4 | FKBP4 | IPI00219005.3 | 2288 | 3.05 | 3.464 |
| Isocitrate dehydrogenase 1 [NADP+] | IDH1 | IPI00011107.2 | 3417 | 8.63 | 3.138 |
| Electron transfer flavoprotein subunit beta | ETFB | IPI00004902.1 | 2109 | 16.08 | 3.071 |
| NADH dehydrogenase (ubiquinone) 1 | NDUFAB1 | IPI00022442.2 | 4706 | 8.97 | 2.752 |
| Single-stranded DNA-binding protein 1 | SSBP1 | IPI00029744.1 | 6742 | 23.65 | 2.747 |
| Malate dehydrogenase 2 | MDH2 | IPI00291006.2 | 4191 | 55.62 | 2.678 |
| Uncharacterized protein | ------ | IPI00917605.1 | ----- | 24.75 | 2.600 |
| Cytoplasmic of Fumarate hydratase | FH | IPI00759715.1 | 2271 | 4.50 | 2.575 |
| Proteasome subunit beta type-4 | PSMB4 | IPI00555956.2 | 5692 | 8.33 | 2.549 |
| Tubulin tyrosine ligase-like family, member 12 | TTLL12 | IPI00879002.1 | 23170 | 1.57 | 2.503 |
| Myosin VI | MYO6 | IPI00552641.2 | 4646 | 11.17 | 2.499 |
| Acetyl-CoA acetyltransferase 1 | ACAT1 | IPI00062003.1 | 38 | 5.56 | 2.454 |
| ATPase family, AAA domain containing 3A | ATAD3A | IPI00455487.5 | 55210 | 5.32 | 2.446 |
| Brain type mu-glutathione S-transferase | GSTM3 | IPI00845493.1 | 2947 | 26.67 | 2.432 |
| Dihydrolipoyl dehydrogenase | DLD | IPI00909143.1 | 1738 | 4.12 | 2.427 |
| Electron transfer flavoprotein subunit alpha | ETFA | IPI00895865.1 | 2108 | 26.76 | 2.417 |
| Phosphoenolpyruvate carboxykinase 2 | PCK2 | IPI00797038.1 | 5106 | 1.72 | 2.411 |
| Creatine kinase | CKB | IPI00022977.1 | 1152 | 22.05 | 2.403 |
| Prohibitin | PHB | IPI00017334.1 | 5245 | 29.78 | 2.400 |
| Uncharacterized protein | ------ | IPI00916763.1 | ----- | 31.68 | 2.356 |
| Peptidyl-prolyl cis-trans isomerase B | PPIB | IPI00646304.4 | 5479 | 18.98 | 2.337 |
| Enoyl-CoA hydratase, peroxisomal | ECH1 | IPI00011416.2 | 1891 | 13.11 | 2.320 |
| Creatine kinase U-type, mitochondrial 1B | CKMT1B | IPI00658109.1 | 1159 | 10.07 | 2.296 |
| Prohibitin 2 | PHB2 | IPI00797822.2 | 11331 | 25.29 | 2.280 |
| Heat-shock protein 105 kDa | HSPH1 | IPI00908988.1 | 10808 | 4.28 | 2.270 |
| Aldo-keto reductase family 1 | AKR1A1 | IPI00220271.3 | 10327 | 8.92 | 2.269 |
| Enoyl-CoA hydratase, mitochondrial | ECHS1 | IPI00024993.4 | 1892 | 12.07 | 2.184 |
| Alpha-ketoglutarate-dependent dioxygenase | FTO | IPI00845224.1 | 79068 | 31.67 | 2.138 |
| Mitochondrial outer membrane fission 1 protein | FIS1 | IPI00007052.6 | 51024 | 7.24 | 2.121 |
| Tyrosine 3-monooxygenase activation protein, gamma | YWHAG | IPI00220642.7 | 7532 | 39.27 | 2.102 |
| Voltage-dependent anion-selective channel protein 1 | VDAC1 | IPI00216308.5 | 7416 | 17.31 | 2.101 |
| Mitochondrial carrier 2 | MTCH2 | IPI00003833.3 | 23788 | 4.62 | 2.093 |
| ADP/ATP translocase 2 | SLC25A5 | IPI00007188.5 | 292 | 20.47 | 2.087 |
| Hemoglobin alpha 2 | HBA2 | IPI00853068.2 | 3040 | 8.18 | 2.084 |
| D-dopachrome tautomerase | DDT | IPI00893737.1 | ---- | 22.00 | 2.080 |
| Peroxiredoxin 2 | PRDX2 | IPI00909207.1 | 7001 | 24.04 | 2.075 |
| Cytosolic non-specific dipeptidase 2 | CNDP2 | IPI00177728.3 | 55748 | 7.79 | 2.044 |
| Heat shock 60 kDa protein 1 (chaperonin) | HSPD1 | IPI00784154.1 | 3329 | 38.22 | 2.040 |
| Cytochrome c oxidase subunit IV isoform 1 | COX4I1 | IPI00645361.1 | 1327 | 14.46 | 2.034 |
