# Supplementary material for: Inactivation of ID4 promotes a CRPC phenotype with constitutive AR activation through FKBP52
Source: Mol Oncol. 2017 Mar 2;11(4):337–57. doi: 10.1002/1878-0261.12028 (PMC5378613; doi:10.1002/1878-0261.12028)
Supplement: Supplementary file 6 — Table S3. List of significantly down‐regulated proteins in L(−)ID4 compared with those in L+ns cells. [file MOL2-11-337-s006.docx]

| **Protein Name** | **Symbol** | **Accession #** | **Gene ID** | **Coverage** | **Fold Change** |
| --- | --- | --- | --- | --- | --- |
| Fascin actin-bundling protein 1 | FSCN1 | IPI00163187.10 | 6624 | 5.68 | 0.489 |
| Golgi glycoprotein 1 | GLG1 | IPI00641153.3 | 2734 | 1.53 | 0.485 |
| Hypoxanthine Phosphoribosyltransferase 1 | HPRT | IPI00218493.7 | 3251 | 4.59 | 0.476 |
| NAD(P)H dehydrogenase, quinone 1 | NQO1 | IPI00910902.1 | 1728 | 38.12 | 0.466 |
| Myoferlin | MYOF | IPI00216268.1 | 26509 | 2.23 | 0.459 |
| Integrin alpha-3 | ITGA3 | IPI00965926.1 | 3675 | 1.44 | 0.456 |
| 36 kDa uncharacterized protein | ---- | IPI00872379.1 | ---- | 39.50 | 0.453 |
| Calpain-2 | CAPN2 | IPI00910745.1 | 824 | 8.99 | 0.441 |
| Acidic (leucine-rich) nuclear phosphoprotein 32 family, member E | ANP32E | IPI00165393.1 | 81611 | 24.63 | 0.430 |
| Actinin, alpha 1 | ACTN1 | IPI00909239.1 | 87 | 18.71 | 0.421 |
| Integrin | ITGB1 | IPI00293305.4 | 3688 | 6.08 | 0.420 |
| Collagen-binding protein 2 | SERPINH1 | IPI00910487.1 | 871 | 6.55 | 0.410 |
| 68 kDa uncharacterized protein | ---- | IPI00872814.1 | ---- | 11.63 | 0.409 |
| Lectin, galactoside-binding, soluble, 1 | LGALS1 | IPI00219219.3 | 3956 | 17.78 | 0.403 |
| Keratin 19 | KRT19 | IPI00306959.11 | 3880 | 34.33 | 0.398 |
| Clathrin light chain A | CLTA | IPI00216393.1 | 1211 | 7.34 | 0.393 |
| 11 kDa uncharacterized protein | ---- | IPI00878392.1 | ---- | 11.58 | 0.382 |
| Thioredoxin-related transmembrane protein 1 | TMX1 | IPI00395887.4 | 81542 | 8.93 | 0.365 |
| Keratin 19 | KRT19 | IPI00479145.3 | 3880 | 9.50 | 0.345 |
| Uncharacterized protein | ---- | IPI00966258.1 | ---- | 34.30 | 0.344 |
| Annexin A1 | ANXA1 | IPI00218918.5 | 301 | 32.08 | 0.330 |
| Thymosin beta 4, x-linked pseudogene 8 | TMSL3 | IPI00180240.2 | 7117 | 15.91 | 0.329 |
| Annexin A2 | ANXA2 | IPI00455315.4 | 302 | 60.77 | 0.328 |
| LIM domain 7 | LMO7 | IPI00552510.2 | 4008 | 1.04 | 0.300 |
| S100 calcium binding proteins A11 | S100A11 | IPI00013895.1 | 6282 | 32.38 | 0.287 |
| Glutaminase | GLS | IPI00215687.1 | 2744 | 7.86 | 0.270 |
| Nucleoredoxin | NXN | IPI00908739.1 | 64359 | 15.87 | 0.269 |
| Ezrin | EZR | IPI00843975.1 | 7430 | 4.44 | 0.266 |
| Transglutaminase 2 | TGM2 | IPI00294578.1 | 7052 | 14.56 | 0.228 |
| Vimentin | VIM | IPI00418471.6 | 7431 | 43.56 | 0.227 |
| Stratifin | SFN | IPI00013890.2 | 2810 | 40.32 | 0.219 |
| Hippocalcin | HPCA | IPI00219103.6 | 3208 | 4.15 | 0.209 |
| S100 calcium binding protein A6 | S100A6 | IPI00027463.1 | 6277 | 38.89 | 0.136 |
| Cysteine-rich protein 1 | CRIP1 | IPI00215611.5 | 1396 | 9.09 | 0.055 |
