# Supplementary material for: Inactivation of ID4 promotes a CRPC phenotype with constitutive AR activation through FKBP52
Source: Mol Oncol. 2017 Mar 2;11(4):337–57. doi: 10.1002/1878-0261.12028 (PMC5378613; doi:10.1002/1878-0261.12028)
Supplement: Supplementary file 7 — Appendix S1. Antibodies and reagents for Immunoblots, co‐immunoprecipitation, Immunocytochemistry, Immunohistochemistry, and ChIP assays. Appendix S2. List of real‐time PCR primer sequences. Appendix S3. List of quantitative RT‐PCR primer sequences used for ChIP analysis. [file MOL2-11-337-s007.docx]

**1.1. Antibodies and reagents for Immunoblots, Co-immunoprecipitation, Immunocytochemistry, Immunohistochemistry, and ChIP assays**

|  | | |
| --- | --- | --- |
| **Protein** | **Company Antibodies** | **Cat. Number** |
| ID4 | Biocheck | BCH-9/82-12 |
| AR | EMD Millipore | 06-680 |
| ARD1 | Santa Cruz Biotechnology | Sc-33820 |
| PSA | Dako Technologies | A0562 |
| FKBP51 | Dr Marc Cox Laboratory | ------ |
| FKBP52 | Dr Marc Cox Laboratory | ------ |
| Hsp90 | Proteintech | 13171-1-AP |
| Hsp27 | Cell Signalling | #2402 |
| P-Hsp27 | Enzo Life Sciences | ADI-SPA-524 |
| KI67 | Abcam | Ab15580 |
| LAMIN A | Abcam | Ab108595 |
| GAPDH | Cell Signalling | #5174 |
| TOPO I | Santa Cruz Biotechnology | Sc-271285 |
| Pierce Goat Anti-Rabbit Poly-HRP | Thermo Scientific | 32260 |
| Pierce Goat Anti-Mouse Poly-HRP | Thermo Scientific | 32230 |
| **ICC secondary antibodies and reagents** |  |  |
| DyLight 594 goat anti-mouse (red) | Thermoscientific | #35510 |
| DyLight 488 goat anti-rabbit (green) | Thermoscientific | #35552 |
| DyLight 594 goat anti-rabbit (red) | Thermoscientific | #35560 |
| DyLight 488 goat anti-mouse (green) | Thermoscientific | #35502 |
| DAPI (Pro-long Anti-fade) | Invitrogen | #P36935 |

**1.2. List of Real-time PCR Primer Sequences**

| **Gene** | **5'Primer** | **3'Primer** |
| --- | --- | --- |
| AR | 5'GAAGCCATTGAGCCAGGTGT3' | 5'TCGTCCACGTGTAAGTTGCG3' |
| ARD1 | 5'GGAGAGCAAAGGCAATTCA3' | 5'CCTCTGAGCTGTCCTTGACA3' |
| FKBP51 | 5'TTCCCTCGAATGCAACTCTC3' | 5'TCTACTGTTGCTCCTTCGTTTG3' |
| FKBP52 | 5'AGCCCCAAACAGGACGAA3' | 5'AGTGTAGTGGACAAAGACTCGG3' |
| GAPDH | 5'GAAGGTGAAGGTCGGAGTC3' | 5' GAAGATGGTGATGGGATTTC 3' |
| HSP27 | 5'TCCCTGGATGTCAACCACTTCG3' | 5'GGGACAGGGAGGAGGAAACTTG3' |
| PSA | 5'GCTGTGAAGGTCATGGACCT3' | 5'CCAGCACACAGCATGAACTT3' |

**1.3. List of Quantitative RT-PCR Primer Sequences used for ChIP Analysis**

| **Gene** | **5'Primer** | **3'Primer** |
| --- | --- | --- |
| ETV1 | 5'TTTTGTGAATGGGACTGTCG3' | 5'AGGGGAACAAGATGGCTTTT3' |
| FKBP51 | 5'GGAGCCTCTTTCTCAGTTTTG3' | 5'CAATCGGAGTGTAACCACATC3' |
| PSA | 5'CATGTTCACATTAGTACACCTTGCC3' | 5'TCTCAGATCCAGGCTTGCTTACTGTC3' |
| TMPRSS2 | 5'TGGTCCTGGATGATAAAAAAAGTTT3' | 5'GACATACGCCCCACAACAGA3' |
