# Supplementary material for: Inactivation of ID4 promotes a CRPC phenotype with constitutive AR activation through FKBP52
Source: Mol Oncol. 2017 Mar 2;11(4):337–57. doi: 10.1002/1878-0261.12028 (PMC5378613; doi:10.1002/1878-0261.12028)
Supplement: Supplementary file 8 [file MOL2-11-337-s008.docx]

***Supplementary Information – Table Legends***

Supplementary Table 1 - ID4 binding partners identified from prostate cancer LNCaP cells using a two-step co-immunoprecipitaton and mass spectrometry approach.

Supplementary Table 2 - List of Significantly Up-Regulated Proteins in L(-)ID4 Compared with Those in L+ns cells.

Supplementary Table 3 **-** List of Significantly Down-Regulated Proteins in L(-)ID4 Compared with Those in L+ns cells.

***Supplementary Information – Figure Legends***

Supplementary Figure 1 - Expression of ID4 correlates inversely with major AR-regulated expression in prostate cancer. ID4, AR and AR-dependent gene expression including PSA, FKBP51, and ARD1 in prostate adenocarcinoma (PCa, blue) as compared to adjacent normal prostate (ANP, Pink) in The Cancer Genome Atlas (TCGA) prostate cancer adenocarcinoma (PRAD) gene expression (Illumina Hiseq) database.

Supplementary Figure 2 - Co-localization of AR and P-Hsp27 in L+ns and L(-)ID4 cell lines. Immunofluorescence of AR (red) and P-Hsp27 [Ser82] (green) in L+ns and L(-)ID4 cells in the absence or presence of R1881 (10 nM) for 24 hours. Red and green staining is protein specific and blue is nuclear (DAPI) (see respective insets). A representative image of 3 different experiments is shown.

Supplementary Figure 3 - Schematic model of ID4 regulated AR activity in prostate cancer cells. Data presented in this study support the predicted model implicating the pivotal role of FKBP52 in potentiating AR signaling, following the loss of ID4 in prostate cancer LNCaP cells [L(-)ID4].
